# Aberrant Functional and Causal Connectivity in Acute Tinnitus With Sensorineural Hearing Loss

**DOI:** 10.3389/fnins.2020.00592

**Published:** 2020-06-30

**Authors:** Yuexin Cai, Mingwei Xie, Yun Su, Zhaopeng Tong, Xiaoyan Wu, Wenchao Xu, Jiahong Li, Fei Zhao, Caiping Dang, Guisheng Chen, Liping Lan, Jun Shen, Yiqing Zheng

**Affiliations:** ^1^Department of Otolaryngology, Sun Yat-sen Memorial Hospital, Sun Yat-sen University, Guangzhou, China; ^2^Institute of Hearing and Speech-Language Science, Sun Yat-sen University, Guangzhou, China; ^3^Department of Radiology, Sun Yat-sen Memorial Hospital, Sun Yat-sen University, Guangzhou, China; ^4^Department of Speech and Language Therapy and Hearing Science, Cardiff Metropolitan University, Cardiff, United Kingdom; ^5^Department of Hearing and Speech Science, Xinhua College, Sun Yat-sen University, Guangzhou, China; ^6^Affiliated Brain Hospital of Guangzhou Medical University, Guangzhou, China; ^7^Department of Psychology, Guangzhou Medical University, Guangzhou, China

**Keywords:** acute tinnitus, hearing loss, functional magnetic resonance imaging (fMRI), functional connectivity, causal connectivity

## Abstract

**Purpose:**

The neural bases in acute tinnitus remains largely undetected. The objective of this study was to identify the alteration of the brain network involved in patients with acute tinnitus and hearing loss.

**Methods:**

Acute tinnitus patients (*n* = 24) with hearing loss and age-, sex-, education-matched healthy controls (*n* = 21) participated in the current study and underwent resting-state functional magnetic resonance imaging (fMRI) scanning. Regional homogeneity and amplitude of low-frequency fluctuation were used to investigate the local spontaneous neural activity and functional connectivity (FC), and Granger causality analysis (GCA) was used to analyze the undirected and directed connectivity of brain regions.

**Results:**

Compared with healthy subjects, acute tinnitus patients had a general reduction in FC between auditory and non-auditory brain regions. Based on FC analysis, the superior temporal gyrus (STG) revealed reduced undirected connectivity with non-auditory brain regions including the amygdala (AMYG), nucleus accumbens (NAc), the cerebellum, and postcentral gyrus (PoCG). Using the GCA algorithm, increased effective connectivity from the right AMYG to the right STG, and reduced connectivity from the right PoCG to the left NAc was observed in acute tinnitus patients with hearing loss. The pure-tone threshold was positively correlated with FC between the AMYG and STG, and negatively correlated with FC between the left NAc and the right PoCG. In addition, a negative association between the GCA value from the right PoCG to the left NAc and the THI scores was observed.

**Conclusion:**

Acute tinnitus patients have aberrant FC strength and causal connectivity in both the auditory and non-auditory cortex, especially in the STG, AMYG, and NAc. The current findings will provide a new perspective for understanding the neuropathophysiological mechanism in acute tinnitus.

## Introduction

Tinnitus is defined as the perception of a phantom sound in the absence of an internal or external sound source (Chen et al., 2017c; Cai et al., 2018; Vanneste et al., 2019). The prevalence of tinnitus reaches about 10–15% of the adult population. Of these, about 10–20% of patients are affected severely and sought medical therapy (Cai et al., 2017; Yang et al., 2018). At present, the clinical treatment for tinnitus includes counseling, sound therapy, cognitive behavioral therapy, etc., however, no identified treatments have been effective and unequivocal to all types of tinnitus (Chen et al., 2017b; Tsai et al., 2018). One of the most important reasons is that the mechanism of tinnitus remains unclear. Nowadays, it is widely accepted that tinnitus in not only caused by peripheral cochlear impairment, but also the aberrant activity in the central nervous system (Chen et al., 2017b; Cai et al., 2018). A large number of neuroimaging studies have reported that abnormality of central activities are involved in both auditory and non-auditory brain regions for chronic tinnitus (Chen et al., 2017c; Vanneste et al., 2019).

In auditory cortices, several studies revealed that this brain region, including the middle temporal gyrus (MTG) and superior temporal gyrus (STG), predominantly had hyperactivity in patients with chronic tinnitus (Chen et al., 2017c; Han et al., 2018). Middle temporal gyrus has been proven to be a key region of the default mode network (DMN), whose activity might depend on people’s attention or goal-directed behavior (Raichle et al., 2001; Mantini et al., 2007). The perception of a phantom auditory sensation from tinnitus might lead to its hyperfunction. In addition, the hyperactivity of STG has been considered to reflect the auditory phantom phenomenon (Smits et al., 2007). In non-auditory brain regions, especially the limbic system, altered neural activity has been regarded as neural decompensation for the tinnitus auditory phantom. Equivalent to the descending pain inhibitory system, a similar “noise canceling” system mainly consisting of the nucleus accumbens (NAc) and the ventromedial prefrontal cortex (vmPFC) has been evidenced and found in studies using both anatomical and functional magnetic resonance imaging (fMRI). Rauschecker et al. (2015) showed disrupted core areas of these regions, which consequently, might cause the occurrence or/and persistence of tinnitus (Rauschecker et al., 2015). In addition, Song et al. (2015) demonstrated that the neural activity of rostral anterior cingulate cortex (ACC) and the connectivity between primary auditory cortex and ACC had a significantly negative correlation with the tinnitus awareness grade. As a result, the authors suggested that rostral ACC is likely to become another core area of the noise canceling system in response to the perception of tinnitus (Song et al., 2015).

Furthermore, many pathophysiological models have also been developed to explain the mechanism of chronic tinnitus, including central gain, neural synchrony, frontostriatal gating, noise canceling deficits, and global workspace (Vanneste et al., 2011; Hashmi et al., 2013; Elgoyhen et al., 2015; Rauschecker et al., 2015; Wallhäusser-Franke et al., 2017; Cai et al., 2018; Zhou et al., 2019). However, few studies have investigated central neural processing during an acute period, thus, the mechanism of acute tinnitus remains unclear. It is therefore crucial to clarify the alteration of the brain network of acute tinnitus to prevent its development from acute to chronic tinnitus. The abnormalities in the auditory and non-auditory brain regions found in patients with chronic tinnitus could provide important clues for further research of acute tinnitus.

A recent study by Zhou et al. (2019) investigated the alterations of brain function using resting-state functional magnetic resonance imaging (rs-fMRI) in 28 patients with unilateral acute tinnitus and hearing loss. Abnormal neural activity and altered connectivity in both the auditory and non-auditory cortex were found. The disrupted cortex regions included increased activity in the right temporal gyrus and decreased in the cerebellar vermis, the right calcarine cortex, the right precuneus, the right supramarginal gyrus (SMG), as well as the right middle frontal gyrus (MFG) in acute tinnitus, which associated with the DMN, attention network, visual network, and executive control network (Zhou et al., 2019). In a recent electroencephalography (EEG) study, reduced activity in the left frontal cortex, decreased connectivity between the temporal gyrus and the SMG, as well as the central cingulate gyrus and frontal gyrus in a comparison between 25 acute tinnitus subjects and 27 healthy controls, was shown (Cai et al., 2019). These studies support the aberrant central changes in brain activity in tinnitus during the acute period, however, the findings from these studies are inconsistent and the causal connectivity in neural networks in acute tinnitus remains largely undetected.

Tinnitus is usually associated with sensorineural hearing loss (HL). Sensorineural hearing loss has been identified as the most relevant factor to trigger tinnitus. About 75% of unilateral tinnitus and over 80% of bilateral tinnitus patients have a HL in the standard audiogram detection with thresholds exceeding 20 dB (Wallhäusser-Franke et al., 2017). Studies on acute tinnitus are usually concerned with tinnitus following idiopathic sudden sensorineural hearing loss (ISSNHL). Idiopathic sudden sensorineural hearing loss with tinnitus is a good candidate for the investigation of the pathophysiological mechanism of acute tinnitus (Mühlmeier et al., 2016; Wallhäusser-Franke et al., 2017).

Resting-state fMRI has been widely used to detect intrinsic neuro-pathophysiological mechanisms in terms of aberrant neural activity and brain functional connectivity (FC) changes in various neurological related disorders, such as chronic tinnitus, Alzheimer’s disease, and Parkinson (Chen et al., 2018b,c,d). Resting-state fMRI measurement is based on the low-frequency (0.01–0.1 Hz) fluctuations in blood-oxygenation-level dependent (BOLD) signals, and thus regional homogeneity (ReHo) can be applied to demonstrate abnormal neural coherence across the whole brain, by quantifying the local synchronization within adjacent voxels during resting state (Zang et al., 2004). With regards to this measurement, amplitude of low-frequency fluctuation (ALFF) is widely regarded as an effective approach that measures intrinsic neural response at the sing-voxel level to reflect the intensity of regional neuronal spontaneous activity (Biswal et al., 1995). Moreover, fractional ALFF (fALFF) is a better approach to detect the resting-state regional spontaneous neural activity in comparison to ALFF analysis, due to the reduction of physiological noise (Zou et al., 2008).

Functional connectivity is an effective method to explore the synchronization of functional activities in different brain regions as well as the correlations between them, while Granger causality analysis (GCA) is explicitly used to examine the functional relationship in a directed manner (Friston et al., 2013), emphasizing the causality between brain regions. The purpose of this study is therefore, to investigate the aberrant functional and causal connectivity of central neural regions in patients with acute tinnitus, by combining FC and GCA analysis. It is hypothesized that alterations of the central neural network can reveal the central neural mechanisms associated with the characteristics of acute tinnitus.

## Materials and Methods

### Participants

In this study, 28 right-handed ISSNHL patients with tinnitus, and 23 age- and gender-matched healthy subjects were recruited from the Ear, Nose, and Throat clinic, Sun Yat-sen Memorial hospital, Guangzhou, China, through recruitment advertisements. The selection criteria for ISSNHL subjects with tinnitus were as follows:

• Patients had no less than 30 dB sensorineural hearing impairment associated with tinnitus in more than three consecutive audiometric frequencies that had onset within 3 days.
• The duration of patients’ relevant symptom was less than 1 month.
• Patients had sought clinical help due to hearing impairment and tinnitus.
• Subjects with middle ear surgery, pulsatile tinnitus, Ménière’s disease, autoimmune hearing loss, acoustic neurinoma, central nervous system disorders, head trauma, depression, insomnia or magnetic resonance imaging (MRI) contraindications were excluded.
• Subjects with a head motion greater than 2.0 mm translation in any plane or a 2.0° rotation in any direction during MRI scanning were excluded.

The hearing thresholds of all participants were determined by pure tone audiometry (PTA) examination. Subjects were asked to sit in an acoustically shielded room for the test. Air conduction thresholds at 0.125, 0.25, 0.5, 1, 2, 4, and 8 kHz were measured on both ears. Bone conduction thresholds at 0.25, 0.5, 1, 2, and 4 kHz were also determined. Mean hearing threshold was calculated from hearing thresholds at 0.5, 1, 2, and 4 kHz (Audiology, 1988; Cai et al., 2019).

All patients were asked to describe the duration and laterality of tinnitus. Afterward, a tinnitus pitch matching test was performed (Teismann et al., 2011), in which the tinnitus pitch match was determined using the frequencies 0.125, 0.25, 0.5, 1, 2, 3, 4, 6, and 8 kHz. In the process of the test, participants were asked to contrast the perceptive tinnitus pitch and the matching pitch, and the matching pitch was adjusted according to the feedback from the subjects. Ultimately, a more precise or approximate matching pitch was obtained by adjusting the frequency in half-octave steps. If no matching pure tone was gained from subjects, narrow band noise was applied.

Moreover, the tinnitus handicap inventory (THI; Newman et al., 1996), was used to assess the severity of tinnitus and related distress after the tinnitus specific assessment. The duration of education of all subjects were also collected.

Data was collected before patients received any therapeutic intervention, within 30 days, however, four ISSNHL patients with tinnitus and two healthy volunteers were excluded because of excessive head motion during MRI scanning. Therefore, 24 ISSNHL patients with tinnitus (10 males and 14 females; age mean = 43.57 years, SD = 15.42 years) and 21 healthy subjects (12 males and 9 females; age mean = 44.48 years, SD = 17.05 years) were finally included for the data analysis. Among the patients, eight subjects have tinnitus in the right ear, 10 subjects in the left ear, and six subjects in bilateral ears. All participants were informed of the background and purpose of the study and they signed a written consent form. This study was approved by the Ethics Committee of Sun Yat-sen Memorial Hospital, and written informed consent was obtained from each participant.

### MRI Acquisition

Magnetic resonance imaging was performed on a 3.0 T MRI scanner (Ingenia, Philips Medical Systems, Netherlands) with an 8-channel receiver array head coil. Head motion and scanner noise were reduced using foam padding and earplugs. The subjects were required to lie quietly with their eyes closed but not to fall asleep, to avoid thinking of anything in particular, and to avoid head motion during the scan. Functional images were obtained axially using a gradient echo-planar imaging sequence as follows: repetition time (TR) = 2000 ms, echo time (TE) = 30 ms, slices = 33, thickness = 3.5 mm, gap = 0.7 mm, field of view (FOV) = 224 mm × 224 mm, acquisition matrix = 64 × 64, and flip angle (FA) = 90°. The voxel size was 3.5 mm × 3.5 mm × 3.5 mm, and 240 functional volumes were obtained. High resolution T1-weighted, 3D anatomical images were collected followed by the resting scan for normalization with the following parameters: TR/TE = 7/3.2 ms, slices = 192, thickness = 1 mm, gap = 0 mm, FA = 7°, acquisition matrix = 256 × 256, and FOV = 256 mm × 256 mm. The fMRI sequence lasted 8 min and 8 s, and the structural sequence lasted 6 min and 20 s. All scans were acquired with parallel imaging using the sensitivity encoding (SENSE) technique, SENSE factor = 2.

### Data Preprocessing

Resting-State fMRI data were preprocessed using Data Processing & Analysis for Brain Imaging (DPABI; Yan et al., 2016), which is based on Statistical Parametric Mapping (SPM12)^1^ and the MATLAB R2013a platform. The first 10 volumes were discarded, and the remaining 230 consecutive volumes were corrected for the slice time delay. The realignment was performed to evaluate the degree of head motion and any subjects with a head motion greater than 2.0 mm translation in any plane, or a 2.0° rotation in any direction were excluded. Four sudden deafness with tinnitus, patients and two healthy volunteers were excluded because of excessive head motion during scanning. The 3D anatomical image was co-registered to the functional space, and segmented into white matter, gray matter, and cerebrospinal fluid. According to the segmental information, the realigned functional images were normalized to the standard Montreal Neurological Institute (MNI) template (resampling voxel size = 3 mm × 3 mm × 3 mm). In the calculations, we also regressed out nuisance covariates, including head motion profiles (Friston 24-parameter model; Friston et al., 1995), the linear trend signal, white matter signal, and the cerebrospinal fluid signal.

### Regional Homogeneity Analysis

Temporal bandpass filtering (0.01–0.1 Hz) was performed to reduce the effect of high-frequency before the ReHo calculation. For each participant, ReHo map was generated by Kendall’s coefficient of concordance (KCC), which was used to measure the local synchronization of a given voxel with its 26 nearest neighbors. The ReHo value of each voxel was standardized by partitioning the primal value using the global mean ReHo value. The normalized data were then smoothed with an isotropic Gaussian kernel [full width at half maximum (FWHM) = 4 mm] for further statistical analysis.

### Fractional Amplitude of Low-Frequency Fluctuations Analysis

To increase the signal-to-noise ratio of the preprocessed image, spatial smoothing (FWHM = 4 mm) was conducted before the fALFF calculation. The time courses were then converted to the frequency domain with Fast Fourier Transform and the power spectrum for each voxel was evaluated. Finally, the fALFF map was generated by the ratio of the square root of the power spectrum at the low-frequency band (0.01–0.1 Hz) to the entire detectable frequency band.

### Seed-Based Functional Connectivity Analysis

Twenty (10 left, 10 right) 5-mm-radius spherical regions of interest (ROIs), several prominent brain areas identified in the tinnitus literature, were defined as the seed ROIs for seed-based FC analysis (De Ridder et al., 2011, 2014a,b; Rauschecker et al., 2015; Song et al., 2015; Hullfish et al., 2019): the bilateral auditory cortices, NAc, AMYG, parahippocampal gyrus (PHG), pregenual anterior cingulate cortices (pACC), subgenual ACC, and the ventromedial prefrontal cortices (PFC). The specific MNI coordinates of the seed ROIs are shown in Table 1. For each ROI, we analyzed FC between the seed region and the whole brain at the voxel level using Pearson’s correlation analysis, and a FC map was acquired. The FC map was standardized to a *z* score map using Fisher *r* to *z* transfer, and a zFC map was obtained for each subject.

**TABLE 1:** Seed regions of interest, center coordinates in MNI space.

**Regions of interest**	**L/R**	**Center-of mass coordinates**		
		***x***	***y***	***z***
**Auditory cortices**				
M-TG	L	−57	−18	−15
	R	58	−17	−15
STG	L	−62	−23	12
	R	63	−24	12
	L	−46	−29	10
	R	47	−29	10
	L	−56	−25	5
	R	56	−22	3
**Limbic system**				
NAc	L	−10	12	−13
	R	13	11	−13
AMYG	L	−24	−6	−18
	R	24	−6	−18
PHG	L	−26	−36	−10
	R	26	−36	−10
Pregenual ACC	L	−4	38	4
	R	4	38	4
Subgenual ACC	L	−4	26	−10
	R	4	26	−10
Ventromedial PFC	L	−3	44	−8
	R	3	44	−8

*Coordinates are described in MNI coordinates. MNI, Montreal Neurological Institute; MTG, middle temporal gyrus; STG, superior temporal gyrus; NAc, nucleus accumbens; AMYG, amygdala; PHG, paraHippocampal gyrus; ACC, anterior cingulate cortices; PFC, prefrontal cortices; L, left hemisphere; R, right hemisphere.*

### Granger Causality Analysis

The ROI-wise GCA was performed using the REST-GCA in the REST toolbox^2^ to detect the effective connectivity between each pair of brain regions, which presented significant group-difference in the seed-based FC analysis. For each pair, we extracted time series of the brain regions (*x*, *y*). Signed path coefficients were then calculated as a GCA value, including *x→y* and *y→x*, according to Zang et al. (2012) the extended vector AR model for each subject.

### Statistical Analysis

Two-sample *t*-tests were used to test group differences in demographic and clinical data, such as the age, the PTA, the duration, and the THI. The χ^2^-test was used to compare the between-group difference of gender and education (statistical significance set at *p* < 0.05).

For the ReHo map, fALFF map, and seed-based zFC map, we used two-sample *t*-tests to detect the difference between the ISSNHL patients and healthy controls, and threshold-free cluster enhancement (TFCE) with family-wise error (FWE) correction (*p* < 0.05) was used for multiple comparisons (Chen et al., 2018a). Furthermore, two-sample *t*-tests were used for the GCA value to determine the differences between the two groups. The significance level was set at *p* < 0.05.

Pearson’s correlation was applied to investigate the association between the clinical characteristics and the altered FC/GCA in acute tinnitus subjects (*p* < 0.05, uncorrected for zFC; *p* < 0.0063, Bonferroni correction for GCA).

## Results

### Demographic Information

Except for PTA (*p* < 0.05), there was no significant between-group differences in demographic and other clinical information (*p* > 0.05). The specific information of patients and the healthy subjects is shown in Table 2.

**TABLE 2:** Demographic and clinical characteristics of sudden deafness with tinnitus patients and healthy controls.

	**Patients (*n* = 24)**	**Controls (*n* = 21)**	***p* value**
Age (year)	43.57 ± 15.42	44.48 ± 17.05	0.868^a^
Gender (male; female)	10:14	12:9	0.300^b^
Education levels (years)	12.54 ± 3.50	12.61 ± 4.13	0.293^a^
Tinnitus laterality (right/left/bilateral)	8/10/6	N/A	N/A
PTA of right ear	36.96 ± 28.98	15.57 ± 4.52	0.002^a^
PTA of left ear	38.08 ± 25.99	14.52 ± 4.43	< 0.001^a^
Average PTA	36.27 ± 13.84	15.05 ± 4.20	< 0.001^a^
Duration (days)	9.95 ± 7.08	N/A	N/A
THI	36.25 ± 21.75	N/A	N/A

*Values are represented as Mean ± SD. ^*a*^Two-sample *t*-tests. ^*b*^χ^2^-test. PTA, pure tone audiometry; THI, tinnitus handicap inventory; N/A, non-applicable.*

### ReHo Analysis and fALFF Analysis

There was no significant difference in the ReHo value and the fALFF value between ISSNHL patients and the healthy controls in any brain region (*p* > 0.05, TFCE with FWE correction).

### Functional Connectivity Analysis

**FIGURE 1:**
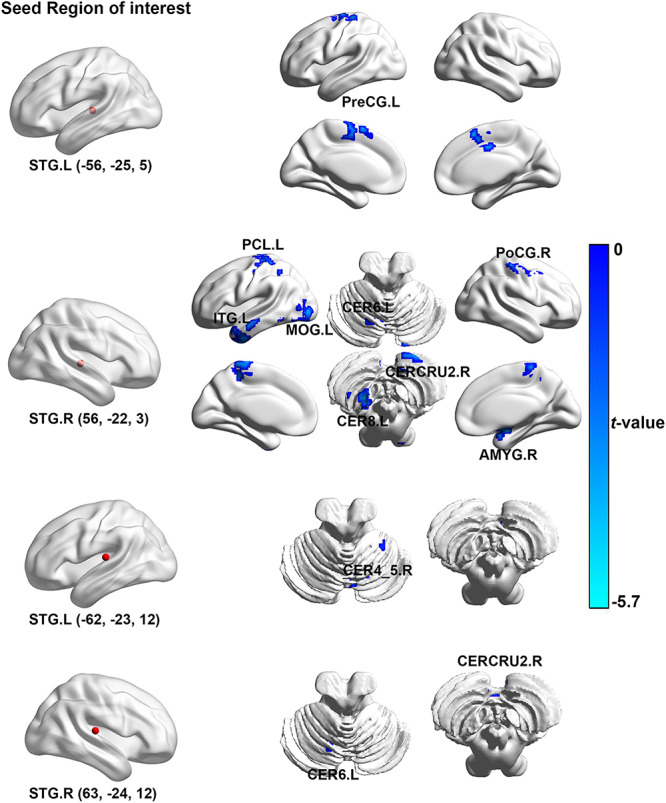
The brain regions with significant difference in the auditory-FC between ISSNHL patients and healthy controls. Left brain diagram shows the seed region of interesting of auditory system, right brain diagram presents the regions with significant group-difference. FC, functional connectivity; STG, superior temporal gyrus; PoCG, postcentral gyrus; MOG, middle occipital gyrus; ITG, inferior temporal gyrus; PCL, paracentral lobule; AMYG, amygdala; CERCRU2, cerebellum_Crus2; CER4_5, cerebellum_4_5; CER6, cerebellum_6; CER8, cerebellum_8; L, left hemisphere; R, right hemisphere. The color bar represents the *t*-value.

**FIGURE 2:**
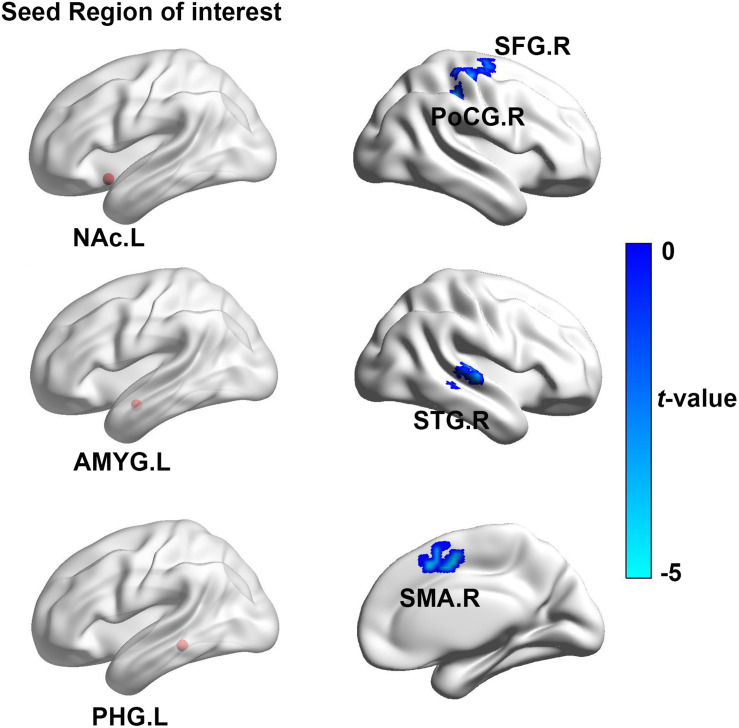
The brain regions show significant difference of the limbic-FC between ISSNHL patients and healthy controls. Left brain diagram shows the seed region of interesting of limbic system, right brain graph presents the regions with significant group-difference. FC, functional connectivity; STG, superior temporal gyrus; PoCG, postcentral gyrus; NAc, nucleus accumbens; SFG, Superior frontal gyrus; AMYG, amygdala; PHG, paraHippocampal gyrus; SMA, supplementary motor area; L, left hemisphere; R, right hemisphere. The color bar represents the *t*-value.

In contrast to the healthy controls, ISSNHL subjects demonstrated prominent decreased FC between the left STG and the right CER4_5 and the left precentral gyrus. In addition, the reduced FC was also observed between the right STG and the right CERCRU2_R, AMYG, postcentral gyrus (PoCG), the left CER8, CER6, MOG, ITG, and the PCL (*p* < 0.05, TFCE with FWE correction; Table 3 and Figure 1). Moreover, in the limbic system, we also detected decreased FC between the left NAc and the right PoCG and the homolateral SFG in ISSNHL patients with tinnitus compared to the healthy subjects. Instead of the NAc, the AMYG, and PHG had similar inhibitions of the FC to other brain regions. Compared with the healthy subjects, the left AMYG had decreased FC to the right STG. The FC also decreased between the left PHG and the right SMA (*p* < 0.05, TFCE with FWE correction; Table 4 and Figure 2).

**TABLE 3:** Regions showing significant differences of auditory-FC between ISSNHL patients and healthy controls.

**Seed region**	**Brain regions**	**Peak MNI coordinates *x*, *y*, *z* (mm)**	**Peak *T* value**	**Voxels**
STG.L (-62, −23, 12)	CER4_5.R	18, −42, −26	−4.83	82
STG.R (63, −24, 12)	CERCRU2.R	3, −78, −39	−4.29	45
	CER6.R	−15, −57, −25	−4.89	45
STG.L (-56, −25, 5)	PreCG.L	−18, −18, 69	−5.59	233
STG.R (56, −22, 3)	CER8.L	−30, −51, −57	−5.15	52
	CER6.L	−12, −66, −24	−4.64	41
	MOG.L	−39, −89, −7	−4.18	267
	ITG.L	−36, 15, −41	−4.27	309
	PCL.L	−9, −27, 72	−4.74	457
	CERCRU2.R	6, −84, −42	−4.36	89
	AMYG.R	24, −3, −15	−4.97	160
	PoCG.R	60, −21, 45	−5.68	188

*MNI, Montreal Neurological Institute; STG, superior temporal gyrus; PoCG, postcentral gyrus; MOG, middle occipital gyrus; ITG, inferior temporal gyrus; PCL, paracentral lobule; AMYG, amygdala; CERCRU2, cerebellum_Crus2; CER4_5, cerebellum_4_5; CER6, cerebellum_6; CER8, cerebellum_8; L, left hemisphere; R, right hemisphere. A corrected threshold of *p* < 0.05 was determined by threshold-free cluster enhancement (TFCE) with family-wise error (FWE) correction.*

**TABLE 4:** Regions showing significant differences of limbic-FC between ISSNHL patients and healthy controls.

**Seed region**	**Brain regions**	**Peak MNI coordinates x, y, z (mm)**	**Peak *T* value**	**Voxels**
NAc.L	PoCG.R	39, −30, 45	−4.78	53
	SFG.R	15, −9, 63	−4.70	28
AMYG.L	STG.R	57, −21, 0	−4.57	33
PHG.L	SMA.R	6, 0, 48	−4.95	57

*MNI, Montreal Neurological Institute; STG, superior temporal gyrus; PoCG, postcentral gyrus; NAc, nucleus accumbens; SFG, superior frontal gyrus; AMYG, amygdala; PHG, paraHippocampal gyrus; SMA, supplementary motor area; L, left hemisphere; R, right hemisphere. A corrected threshold of *p* < 0.05 was determined by threshold-free cluster enhancement (TFCE) with family-wise error (FWE) correction.*

### Granger Causality Analysis

**FIGURE 3:**
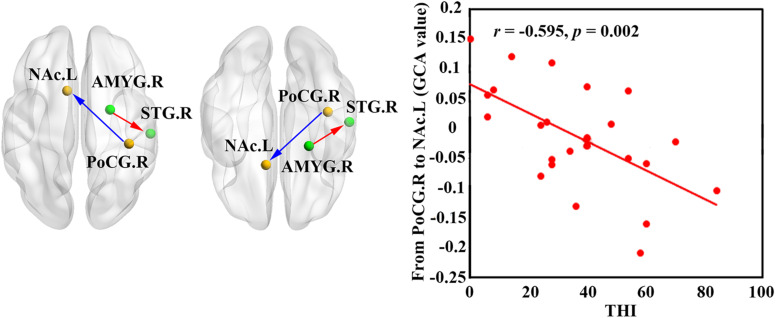
Increased GCA value from AMYG.R to STG.R (in red) and decreased GCA value from PoCG.R to NAc.L (in blue) were found in the ISSNHL patients. The correlations between abnormal GCA value and THI in the ISSNHL patients. The red dots represent each ISSNHL patient. FC, functional connectivity; GCA, granger causality analysis; THI, tinnitus handicap inventory; STG, superior temporal gyrus; AMYG, amygdala; NAc, nucleus accumbens; PoCG, postcentral gyrus; left hemisphere; R, right hemisphere.

According to our seed-based FC analysis results, 16 pairs of brain regions with significant group-differences were selected for the GCA analysis between each pair. In contrast to the healthy controls, patients with ISSNHL demonstrated a significantly increased GCA value from the right AMYG to the right STG (*p* < 0.05; Figure 3). In contrast, a reduced GCA value from the right PoCG to the left NAc was observed in ISSNHL patients (*p* < 0.05; Figure 3).

### Correlation Analysis

**FIGURE 4:**
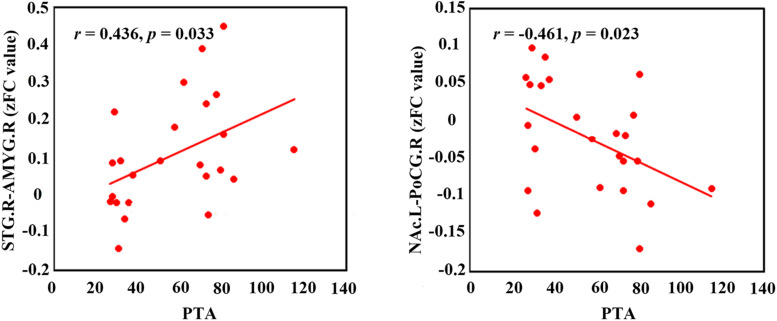
The correlations between the FC with significant group-difference and PTA in the ISSNHL patients. The red dots represent each ISSNHL patient. FC, functional connectivity; PTA, pure tone audiometry; STG, superior temporal gyrus; AMYG, amygdala; NAc, nucleus accumbens; PoCG, postcentral gyrus; left hemisphere; R, right hemisphere.

Figure 4 shows that the PTA was positively correlated with the FC between the right STG and the AMYG (*r* = 0.436, *p* = 0.033). Moreover, the Pearson’s correlation analyses revealed the negative correlation between the PTA and the FC between the left NAc and the right PoCG (*r* = −0.461, *p* = 0.023). In addition, a negative association between the GCA value from the right PoCG to the left NAc and the THI was observed (*r* = −0.595, *p* < 0.002; Figure 3).

## Discussion

This is the first study to use both FC and GCA approaches to explore intrinsic dysfunctional network connectivity related to acute tinnitus. These results highlight the general hypo-connectivity between auditory and non-auditory regions. Based on FC analysis, we found significant decreased connectivity between the STG and several non-auditory regions, including the AMYG, NAc, the cerebellum, and the somatosensory cortex. Using the GCA algorithm, significant increased effective connectivity from the right AMYG to the right STG, and reduced connectivity from the right PoCG to the left NAc was observed in acute tinnitus patients with hearing loss. Moreover, these abnormalities of connectivity were correlated to the intensity of hearing loss and tinnitus distress. Our study clearly demonstrates interregional changes in neural function in acute tinnitus with hearing loss.

### Functional Connectivity and Spontaneous Brain Activation in the STG in Acute Tinnitus With Hearing Loss

The present study showed reduced connections between the STG and associative areas that constitute an emerging feature of brain architecture. Our finding demonstrates that the STG is the main cortical hub in the brain network that is affected by acute tinnitus with hearing loss, which is in line with the hypothesis that the dysconnectivity pattern of the STG is involved in tinnitus perception (Chen et al., 2017a; Qu et al., 2019; Berlot et al., 2020). The STG is regarded as a critical region which receives the neuronal projection from auditory pathway (Binder, 2017) and has been reported to be associated with cognitive functions including memory, auditory cognition and semantics (Jackson et al., 2018). Several neurophysiological models related to STG have been raised as being involved in the acute stage of tinnitus and hearing loss (Fan et al., 2015; Micarelli et al., 2017). In addition, hearing loss may lead to a hypofunction of the auditory cortex which consequently presents a decreased FC between the STG and associative regions. However, a recent study found no significant spontaneous neural activity in the STG and associative areas in ISSNL subjects, which may be due to the short duration of the tinnitus. This finding is in contrast to some previous studies, for example, a PET study reported the decreased FDG uPTAke in the STG, MTG and MFG regions in ISSNHL subjects. The various neuroimaging methods employed, and the tinnitus sample’s heterogeneity may have contributed to the inconsistent results. These results indicated that the short duration of tinnitus accompanying hearing loss, may not necessarily evoke the compensatory enhancement of spontaneous activity, but disrupted FC occurred before the altered local neural changes took place.

Our study also found that non-auditory associative regions reduced connectivity with the STG, which included the cerebellum, PreCG, and the PoCG. The cerebellum is considered as an important hub in detecting auditory afferent and sound processing (Zhou et al., 2019), as well as plays a critical role in a gating control mechanism (Micarelli et al., 2017). Our finding is in line with this previous study, which shows a reduction of cerebellum activity in acute tinnitus. Maudoux et al. also found disrupted connectivity between the auditory cortex and the cerebellum (Maudoux et al., 2012). Therefore, the disrupted connectivity between the cerebellum and STG may induce a reduced capability of contrasting and integrating afferent signals from the auditory cortex, triggering a phantom sound during acute hearing loss.

The PreCG is the anatomical location of the primary motor cortex, which is responsible for controlling voluntary motor movement of the body and involved in the executive functioning of response inhibition. This was consistent with a resting state PET study that patients with SSNHL presented Glucose uPTAke reduced in PreCG and suggested PreCG as a vital region in auditory associated events related to word recognition and phonological processing (Micarelli et al., 2017). The disconnection between PreCG and STG may lead to auditory processing functional decline during the acute stage of tinnitus and hearing loss.

### Functional Connectivity and Correlation Analysis in Limbic System in Acute Tinnitus With Hearing Loss

Tinnitus is often associated with emotional distress and anxiety, these complications may be associated with maladaptive functional connections to the limbic structure, such as the AMYG, hippocampus and the NAc (Hashmi et al., 2013; Lanting et al., 2014; Chen et al., 2017a, c). In this study, reduced undirected connectivity between the AMYG and STG but an increased connectivity between the GCA from the AMYG to the STG was found. The AMYG is a vital part of the subcortical limbic structure, which receives and sends auditory information to the auditory cortex to relay emotional attributes (Yan, 2003; Keuroghlian and Knudsen, 2007; Qu et al., 2019; Xu et al., 2019). Furthermore, the AMYG not only receives information from auditory cortex, but also receives directed projection from subcortical areas of the ascending auditory pathway (Pannese et al., 2016). Therefore, after the acute stage of peripheral auditory deafferentation, the dysfunctional neural synchrony of inter-regions would induce declined connectivity between the AMYG and STG. However, enhanced influence from the AMYG to the STG reflect a central reorganization in the acute period of tinnitus and hearing loss. The AMYG is thought to decode the affective value to the sound perception that is pre-processed, especially in the auditory cortex (Kumar et al., 2012). This is consistent with the findings from previous neuroimaging studies showing that the abnormalities in the AMYG could account for tinnitus and induce chronic depression in tinnitus patients (Micarelli et al., 2017). Activation of the AMYG can augment the central neural plasticity in the auditory cortex (Froemke and Martins, 2011), which could be interpreted as an attempt in conflict monitoring and in informative trace integration coming from sensory error detection, such as those represented by an unexpected auditory input impairment. This heightened influence from the AMYG to the STG might be interpreted as a dysfunctional inhibitory response, directing attention to a phantom sound perception, which may be the possible reason of induced tinnitus.

An interesting finding is the positive correlation between the PTA and FC of the AMYG and STG, which highlight the theories of the compensatory effect of the emotional limbic network. These theories indicate that more intensive hearing loss may be concurrent with more emotional flow from the limbic system to the auditory cortex. However, no significant correlation was observed between the THI scores and the increased influence from the AMYG to the STG, which may be due to the fact that most of the tinnitus subjects presented with lower scores in the THI tests.

Moreover, reduced FC between the PoCG to the NAc and effective connectivity from the PoCG to the NAc was found for ISSNHL with tinnitus. The NAc and associated paralimbic areas play an important role in long-term habituation of continuous unpleasant sounds as well as engaging in the emotional and reward circuit (Hashmi et al., 2013; Lanting et al., 2014). Furthermore, the PoCG belongs to the vital part of somatosensory system. The balance of auditory and somatosensory integration would be altered after loss of auditory input (Froemke and Martins, 2011), which is in line with our finding of a reduced FC between the STG and PoCG in the acute stage of tinnitus and hearing loss. Therefore, besides peripheral auditory system damage, such somatosensory dysfunction may be involved in tinnitus (Job et al., 2012). Previous studies have demonstrated that tinnitus and pain shared a common neuropathological pattern, which suggests a role of the autonomous sympathetic system in tinnitus (Job et al., 2004, 2012). The auditory information was directed *via* the somatosensory system to the NAc for evaluation of the sound’s emotional content. The NAc was also the core region of the noise canceling system (Song et al., 2015). This system leads to the filtering out of unwanted sounds, which then do not reach conscious perception in the auditory cortex. In addition, the FC between the PoCG and NAc was negatively correlated with PTA and also had negative correlations between THI scores and effective connectivity from the PoCG to NAc, indicating that the disruption of information flow from the somatosensory system to the NAc may comprise the noise canceling system after intensive peripheral auditory deafferentation. Consequently the abnormal sound may be passed on to the cortex, causing the conscious perception of tinnitus in ISSNHL patients.

In addition, the NAc is the vital region of brain rewarding circuitry, which plays an important role in discerning and reacting to rewarding and aversive stimuli. As a result, it regulates emotional and cognitive behavior (Russo and Nestler, 2013). ln the brain reward circuitry, the ventral tegmental area with dopaminergic neurons projected to the NAc is thought to “gate” the flow of rewarding information from limbic afferents to basal ganglia outputs. In numerous studies of depression, the NAc contributes neural substrate with the inability to experience pleasure and engage in rewarding activities (Pizzagalli et al., 2009; Russo and Nestler, 2013). Therefore, in association with the reduced connectivity between the PoCG and NAc, the current results indicate that the rewarding pathway dysfunction may underlie a pathophysiology of tinnitus associated depression.

## Limitation

It should be noted that this study has several limitations. This is a cross-sectional study with a limited sample size. In addition, acute tinnitus patients have high heterogeneity in terms of laterality, hearing loss, and related symptoms. It is difficult to eliminate the heterogeneous factors completely even when using strict inclusion and exclusion criteria. Therefore, we should interpret the current results with caution. More extensive and longitudinal investigation with a larger sample size and more subgroups is needed to explore the changes of the directed and undirected FC in a follow-up study of patients with acute tinnitus. Moreover, even though we attempted to diminish the MRI noise by providing earplugs, the subjects could not be completely prevented from hearing noise during the MRI scan, which may influence the neural activity to a certain degree during resting state measurement. This confounding factor should be taken into consideration for all resting-state fMRI studies.

## Conclusion

In this study, we found an aberrant functional network within auditory and non-auditory regions revealed by FC and GCA presented in acute tinnitus with hearing loss. Acute tinnitus patients showed reduced FC between the STG and cerebellum, limbic system, and the somatosensory cortex. Increased effective connectivity from the right AMYG to the right STG and reduced connectivity from the right PoCG to the left NAc were observed in a causal connectivity analysis. A correlation analysis showed functional changes in multiple neural regions between the auditory and limbic areas associated with the gradual increase in the degree of hearing loss during the acute stage of tinnitus. Alteration in brain functional network architecture will contribute to a better understanding of the neuropathophysiological mechanism in acute tinnitus.

## Data Availability Statement

The raw data supporting the conclusions of this article will be made available by the authors, without undue reservation, to any qualified researcher.

## Ethics Statement

The studies involving human participants were reviewed and approved by The Ethics Committee of Sun Yat-sen Memorial Hospital. The patients/participants provided their written informed consent to participate in this study.

## Author Contributions

YC and MX conceived and designed the study. XW, ZT, and YS performed the experiments. WX, JL, FZ, and CD analyzed the data. GC and LL drafted the manuscript. JS and YZ contributed to the discussion and manuscript revision. All authors had contributions on preparing this manuscript.

## Conflict of Interest

The authors declare that the research was conducted in the absence of any commercial or financial relationships that could be construed as a potential conflict of interest.

## Footnotes

1 http://www.fil.ion.ucl.ac.uk/spm

2 http://www.restfmri.net/forum/
